# Decreases in serum levels of S100A8/9 (calprotectin) correlate with improvements in total swollen joint count in patients with recent-onset rheumatoid arthritis

**DOI:** 10.1186/ar3426

**Published:** 2011-07-26

**Authors:** Lucie Andrés Cerezo, Heřman Mann, Ondřej Pecha, Lenka Pleštilová, Karel Pavelka, Jiří Vencovský, Ladislav Šenolt

**Affiliations:** 1 Institute of Rheumatology, First Medical Faculty, Charles University, Na Slupi 4, 128 50 Prague 2, Czech Republic; 2 Institute of Biophysics and Informatics, First Faculty of Medicine, Charles University, Salmovská 478/1, 120 00 Prague 2, Czech Republic

**Keywords:** rheumatoid arthritis, S100 proteins, disease activity, relapse

## Abstract

**Introduction:**

The aim of this study was to examine the serum levels of S100 proteins and to evaluate their role in patients with recent-onset rheumatoid arthritis (RA).

**Methods:**

Serum levels of S100A8/9 and S100A12 were analysed in 43 patients with recent-onset RA, both before and three months after the initiation of conventional treatment, as well as in 32 healthy individuals. Disease activity was assessed based on serum levels of C-reactive protein (CRP), the Disease Activity Score for 28 joints (DAS28) and the total number of swollen joints count for 66 joints (SJC).

**Results:**

The levels of serum S100A8/9 and S100A12 were significantly higher in patients with recent-onset RA compared to the levels in healthy individuals (*P *< 0.0001) and normalised after three months of treatment. Using age- and sex-adjusted analysis, S100A8/9 levels were correlated with CRP (*r *= 0.439, *P *< 0.01), DAS28 (*r *= 0.501, *P *= 0.002) and SJC (*r *= 0.443, *P *= 0.007), while S100A12 was less significantly correlated with these parameters. Higher levels of S100A8/9 at baseline predicted improvement in the levels of CRP and SJC over time. Moreover, decreases in serum S100A8/9 were associated with decreased serum levels of CRP (*r *= 0.459, *P *= 0.005) and improvements in SJC (*r *= 0.459, *P *= 0.005). In multiple linear regression analyses, decreases in S100A8/9 but not CRP were significant predictors for improvements in SJC (*P *= 0.001).

**Conclusions:**

This study is the first to show normalisation of elevated S100 proteins in patients with recent-onset RA after the initiation of conventional treatment. Therefore, S100A8/9 might potentially be a predictive marker for improvement in the total number of swollen joints in patients in the early phase of RA.

## Introduction

Rheumatoid arthritis (RA) is a chronic inflammatory autoimmune disease characterised by synovitis and joint destruction in which the infiltration of inflammatory cells, the activation of synovial fibroblasts and the production of a wide range of inflammatory mediators play significant roles [1,2]. However, the exact pathological processes involved in the initiation of RA remain incompletely understood. Very early RA is suggested to represent an immunopathologically distinct phase of the disease in which a "window of opportunity" for early drug intervention with the potential to prevent joint damage may exist [3]. Recent studies have shown that the development of established RA in patients in the early stages of the disease can be predicted by using clinical and serological measures [4-6]. Therefore, a better understanding of the pathological mechanisms and biomarkers during this early phase would be an important way to determine possible new therapeutic targets and to tailor therapy to ensure optimal treatment for individual patients.

S100 calcium-binding proteins are multifunctional proteins that are implicated in the regulation of a variety of cellular activities [7]. The most familiar S100 proteins, myeloid-related proteins S100A8/9 (calprotectin) and S100A12 (calgranulin C), have recently been proposed as "alarmins," which are the endogenous molecules that signal the early phase of tissue and cell damage [8]. The S100 proteins are expressed predominantly by neutrophils, monocytes and activated macrophages, and increased S100 levels have been demonstrated in several inflammatory diseases [9]. S100A8/9 and S100A12 are increased locally at sites of inflammation as well as in the circulation of patients with RA [10-13]. Moreover, a tight correlation between S100 proteins and laboratory and clinical markers of disease activity has been demonstrated in patients with different arthritides [13-16]. In addition, S100A8/9 and S100A12 were shown to be decreased locally in synovial tissue as well as in the blood in response to different anti-inflammatory therapies, including TNFα inhibitors, and they were upregulated weeks before relapse became clinically apparent in patients with previously well-controlled disease [16-19]. S100A8/9 was associated with measures of joint damage in one cross-sectional study [20]. More importantly, longitudinal data demonstrated that S100A8/9 was a good prognostic biomarker for long-term radiographic joint progression in patients with established RA [21].

However, S100 proteins have not yet been studied in treatment-naïve RA patients. Therefore, we explored the following: (1) the levels of S100 proteins in patients with recent-onset RA, (2) the effect of conventional treatment on the levels of serum S100 proteins, (3) the association between S100 proteins and disease activity and (4) a potential role of S100 proteins as surrogate predictive markers in a short-term longitudinal study.

## Materials and methods

### Patients and clinical examination

A total of 43 patients with recent-onset RA were included in this study. Inclusion criteria were as follows: (1) age > 18 years, (2) fulfilment of the American College of Rheumatology/European League Against Rheumatism (EULAR) 2010 classification criteria for RA at baseline [22] and (3) symptom duration of less than six months. None of the patients had been receiving disease-modifying antirheumatic drugs (DMARDs) or glucocorticoids (GCs) at baseline. After the initiation of conventional treatment, patients were prospectively followed for three months. Disease activity was assessed based on the Disease Activity Score for 28 joints (DAS28) using the number of swollen and tender joints, erythrocyte sedimentation rate (ESR) and the patient's global assessment of activity on a visual analogue scale (VAS) [23]. Swollen joints count for 66 joints (SJC) was also evaluated. The clinical response was defined by the EULAR response criteria [24]. Patients were characterised as follows: good responders had a DAS28 ≤ 3.2 plus a > 1.2 decrease in DAS28, and moderate responders were defined as having (1) DAS28 ≤3.2 plus a > 0.6 and ≤1.2 decrease in DAS28, (2) DAS28 ≤ 5.1 > 3.2 plus a > 0.6 decrease in DAS28 or (3) DAS28 > 5.1 plus a > 1.2 decrease in DAS28. Nonresponders were defined as having a < 0.6 decrease in DAS28 or a DAS28 > 5.1 plus a ≤1.2 decrease in DAS28. The control group consisted of 32 healthy individuals. The study was approved by the local ethics committee, and informed consent was obtained from all patients prior to initiation of the protocol.

### ELISA

Blood samples were collected from all patients at baseline (before therapy) and three months after the start of therapy. Serum samples were immediately centrifuged and stored at -80°C until analysis. The levels of serum S100A8/9 (Bühlmann Laboratories AG, Schönenbuch, Switzerland) and S100A12 (CycLex Co., Ltd., Nagano, Japan) were measured by using commercially available ELISA kits according to the manufacturers' protocols. Absorbance was detected using the Sunrise ELISA reader (Tecan Group Ltd., Salzburg, Austria) with 450 nm as the primary wavelength. Interassay and intraassay reliability of the S100A8/9 assays were 5.3% and 6.2%, respectively. Interassay and intraassay reliability of the S100A12 assays were 3.3% and 3.4%, respectively. The detection limits were 3 ng/mL for the S100A8/9 assay and 56 pg/mL for the S100A12 assay. C-reactive protein (CRP) was measured using nephelometry. Analyses of serum anticyclic citrullinated peptide antibodies (anti-CCP) and immunoglobulin M rheumatoid factor (IgM-RF) were carried out using standard ELISA kits (Test Line s.r.o., Brno, Czech Republic).

### Statistical analyses

The concentrations of S100 proteins are expressed as means ± SEM. A Kolmogorov-Smirnov test of normality was performed for all variables and their difference scores. Pearson's product-moment correlation coefficients and Spearman's rank correlation coefficients were used in cases of normal and non-normal variables, respectively. When comparing patients and controls, the independent samples *t*-test was used for normal variables and the Mann-Whitney *U *test was used as a nonparametric alternative. Two-way analysis of variance with repeated measures (time × groups) was conducted to determine the differences between groups of patients sorted on the basis of the level of disease activity. In addition, multiple linear regression analysis was performed for differences in scores on the DAS28 and total SJC, and appropriate predictors were chosen using a backward stepwise elimination method. For all statistical evaluations, *P *values below 0.05 were considered to be statistically significant. Statistical analyses were performed using SPSS version 17 software (SPSS Inc., Chicago, IL, USA).

## Results

Table 1 shows the baseline characteristics of the patients and healthy controls. Overall, three patients with recent-onset RA had erosions at baseline. Prior to treatment, 22 patients had highly active disease (mean DAS28 > 5.1), 19 patients had moderate disease activity (mean 3.2 < DAS28 ≤ 5.1) and 2 patients had low disease activity (mean 2.6 ≤ DAS28 < 3.2). Initially, DMARD treatment was started in 42 patients. Thirty-five patients received methotrexate (mean dose at month 3: 14.86 mg/week; range: 7.5 to 20 mg/week), six received sulphasalazine, one received leflunomide and thirty-eight received GCs (prednisone or equivalent; initial mean daily dose: 8.9 mg/day; range: 2.5 to 20 mg/day). After three months of treatment, a significant reduction in disease activity was observed (mean DAS28: 5.3 ± 1.5 to 2.8 ± 1.3; mean SJC: 10.0 ± 9.2 to 1.7 ± 3.6; mean CRP: 16.2 ± 19.8 to 4.2 ± 5.8; *P *< 0.0001 for all comparisons). Thirty-nine patients (90.7%) achieved good or moderate improvement, and eighteen (41.9%) reached remission according to the EULAR response criteria [24].

**Table 1:** Baseline characteristics of patients with recent-onset RA and healthy controls^a^

Characteristics	Recent-onset RA (*n *= 43)	Healthy controls (*n *= 32)
Gender, F/M	30/13	11/21
Mean age, years (±SD)	50.90 ± 16.40	43.97 ± 15.4
Mean CRP, mg/L (±SD)	16.20 ± 20	
Mean ESR, mm/1^st hour ^(±SD)	34.20 ± 23.72	
Mean SJC out of 66 (±SD)	10.00 ± 9.28	
Mean DAS28 score (±SD)	5.31 ± 1.51	
RF positivity, *n *(%)	32 (74.4%)	
Anti-CCP positivity, *n *(%)	22 (51.2%)	
DMARDs/GCs, number of patients	42/35	

^a^Anti-CCP, anticyclic citrullinated peptide antibody; CRP, C-reactive protein; DAS28, Disease Activity Score for 28 joints; DMARDs, disease-modifying antirheumatic drugs; RA, rheumatoid arthritis; ESR, erythrocyte sedimentation rate; F, female; GCs, glucocorticoids; M, male; RF, rheumatoid factor.

### S100 proteins and disease activity at baseline

The levels of serum S100A8/9 correlated significantly with S100A12 at baseline (*r *= 0.845, *P *< 0.0001), an association that became even stronger when adjusted for age and sex (*r *= 0.901, *P *< 0.0001). This positive correlation remained significant three months after the start of the treatment. The levels of S100 proteins were not affected by age or gender (data not shown).

**Figure 1:**
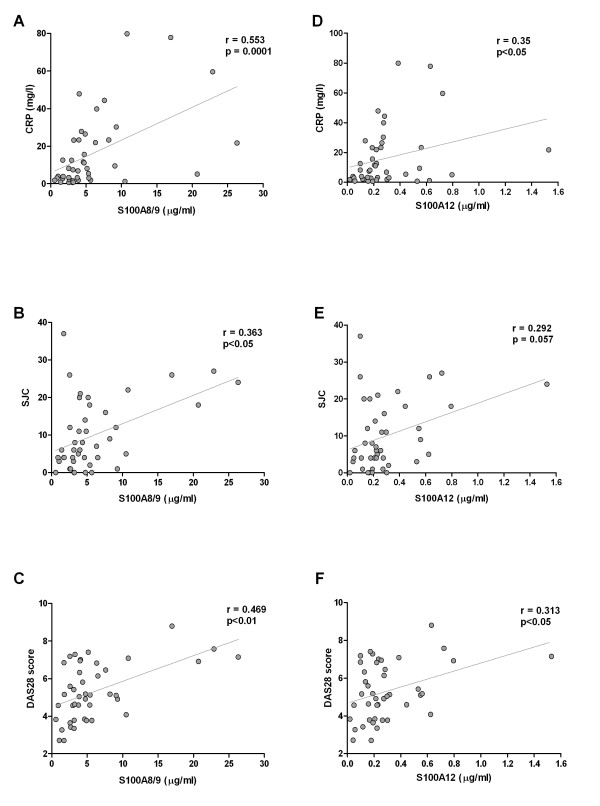
**Scatterplots showing correlations between serum levels of **(A) **through **(C) **S100A8/9 and **(D) **through **(F) **S100A12 and disease activity measures in patients with recent-onset rheumatoid arthritis (RA)**. CRP, C-reactive protein; DAS28, Disease Activity Score for 28 joints; SJC, swollen joint count for 66 joints.

In univariate analysis, the serum levels of S100A8/9 correlated positively with the levels of CRP (*r *= 0.553, *P *< 0.0001), DAS28 (*r *= 0.469, *P *< 0.01) and SJC (*r *= 0.363, *P *< 0.05) at baseline (Figures 1A through 1C). When adjusted for age and sex, these correlations remained significant for CRP (*r *= 0.439, *P *< 0.01) and became stronger for DAS28 (*r *= 0.501, *P *= 0.002) and SJC (*r *= 0.443, *P *= 0.007). Similarly, the levels of S100A12 correlated positively with the levels of CRP (*r *= 0.350, *P *< 0.05) and DAS28 (*r *= 0.313, *P *< 0.05), although there was only a trend for the correlation between the levels of S100A12 and SJC (*r *= 0.292, *P *= 0.057) at baseline (Figures 1D through 1F). When adjusted for age and sex, these correlations were lost for CRP (*r *= 0.288, *P *= 0.083) but remained significant for DAS28 (*r *= 0.354, *P *= 0.034) and became significant for SJC (*r *= 0.345, *P *= 0.039). Furthermore, neither S100A8/9 nor S100A12 was associated with IgM-RF (*r *= -0.056, *P *= 0.727, and *r *= 0.170, *P *= 0.289, respectively) or anti-CCP levels (*r *= -0.195, *P *= 0.210, and *r *= -0.044, *P *= 0.778, respectively).

### Effect of conventional treatment on the levels of S100 proteins

**Figure 2:**
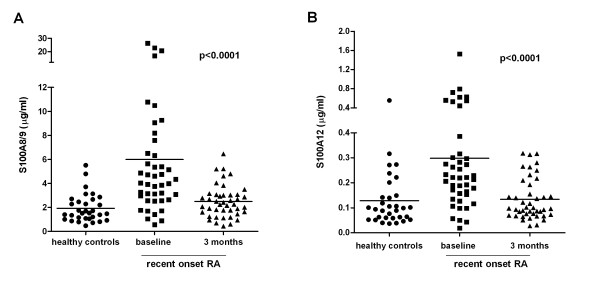
**Serum levels of **(A) **S100A8/9 and **(B) **S100A12 proteins were increased in patients with recent-onset rheumatoid arthritis (RA) and normalised after three months of conventional treatment**. Dots represent healthy controls, squares stand for recent onset RA patients at baseline and triangles represent recent onset RA patients after 3 months treatment.

The levels of serum S100A8/9 (mean 5.99 ± 0.88 μg/mL vs. 1.92 ± 1.16 μg/mL; *P *< 0.0001) and S100A12 (mean 0.30 ± 0.04 μg/mL vs. 0.13 ± 0.11 μg/mL; *P *< 0.0001) were significantly higher in patients with recent-onset RA compared with healthy controls, and these levels essentially normalised after three months of treatment (mean S100A8/9: 5.99 ± 0.88 μg/mL to 2.49 ± 0.21 μg/mL; *P *< 0.0001; mean S100A12: 0.30 ± 0.04 μg/mL to 0.13 ± 0.01 μg/mL; *P *< 0.0001) (Figure 2). Importantly, after three months, the levels of S100A8/9 but not S100A12 were significantly lower in patients who had achieved remission compared with those who showed moderate or high disease activity (mean S100A8/9: 2.15 ± 1.11 μg/mL vs. 3.37 ± 1.34 μg/mL; *P *= 0.043). We found significantly higher levels of baseline S100A8/9 and S100A12 concentrations in patients with active disease compared with those with moderate disease activity (mean S100A8/9: 7.96 ± 1.52 μg/mL vs. 4.20 ± 0.63 μg/mL, *P *= 0.004, and mean S100A12: 0.37 ± 0.07 μg/mL vs. 0.24 ± 0.04 μg/mL, *P *= 0.035, respectively). S100A8/9 and S100A12 serum levels significantly decreased over time, particularly in patients with active disease (mean S100A8/9: 7.96 ± 1.52 μg/mL to 2.81 ± 0.31 μg/mL and mean S100A12: 0.37 ± 0.07 μg/mL to 0.14 ± 0.02 μg/mL; *P *< 0.0001 for both comparisons). However, in patients with moderate disease activity, a modest decrease in S100A12 (mean 0.24 ± 0.04 μg/mL to 0.13 ± 0.02 μg/mL; *P *= 0.046) and a statistically insignificant decrease in S100A8/9 (mean 4.20 ± 0.63 μg/mL to 2.29 ± 0.26 μg/mL; *P *= 0.112) was observed. The levels of S100 proteins were not affected by different dosages of GCs and/or methotrexate.

### Predictive role of S100 proteins

Higher levels of S100A8/9 at baseline predicted an improvement in the level of CRP (*r *= -0.397, *P *= 0.01) and in SJC (*r *= -0.369, *P *< 0.05), but not in DAS28 (*r *= -0.279, *P *= 0.07). When adjusted for age and sex, the correlations of S100A8/9 with changes in the levels of CRP (*r *= -0.423, *P *= 0.01) and changes in SJC (*r *= -0.423, *P *= 0.01) became stronger. The levels of S100A12 at baseline were predictive of the change in SJC (*r *= -0.376, *P *< 0.05), but not in the level of CRP (*r *= -0.212, *P *= 0.184) or in DAS28 (*r *= -0.282, *P *= 0.07), which remained significant when adjusted for age and sex for baseline S100A12 and changes in SJC (*r *= -0.360, *P *= 0.031).

**Figure 3:**
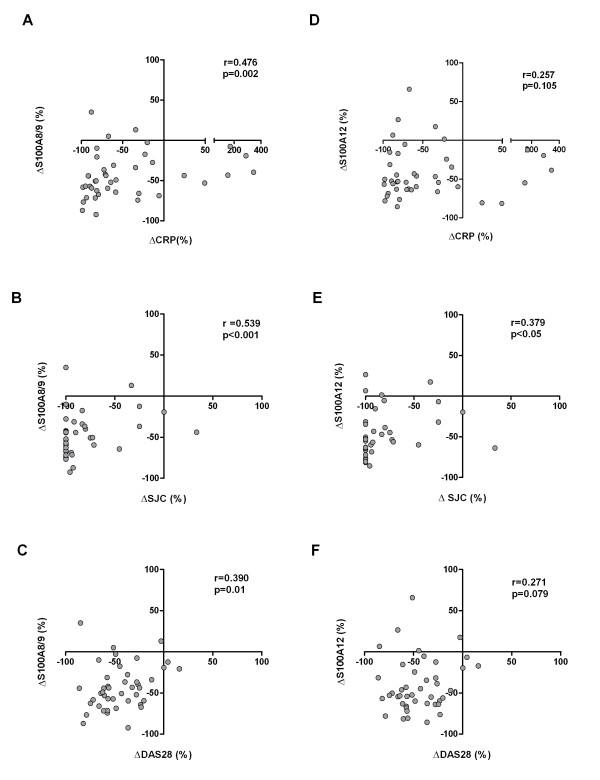
**Scatterplots showing correlations of changes in **(A) **through **(C) **S100A8/9 and **(D) **through **(F) **S100A12 levels during treatment with changes in disease activity measures in patients with recent-onset rheumatoid arthritis**. CRP, C-reactive protein; DAS28, Disease Activity Score for 28 joints; SJC, swollen joint count out of 66.

Furthermore, we found that changes in serum S100A8/9 positively correlated with changes in serum levels of CRP (*r *= 0.476, *P *= 0.002), changes in DAS28 (*r *= 0.390, *P *= 0.01) and changes in SJC (*r *= 0.539, *P *< 0.001) (Figures 3A through 3C). When adjusted for age and sex, the correlations remained significant for changes in CRP (*r *= 0.459, *P *= 0.005) and changes in SJC (*r *= 0.459, *P *= 0.005), but not for changes in DAS28 (*r *= 0.258, *P *= 0.129). However, changes in serum S100A12 correlated with changes in SJC (*r *= 0.379, *P *< 0.05), but not with changes in serum CRP (*r *= 0.257, *P *= 0.105) or changes in DAS28 (*r *= 0.271, *P *= 0.079) (Figures 3D through 3F). Age and sex-adjusted changes in serum S100A12 also correlated only with changes in SJC (*r *= 0.343, *P *= 0.04).

Multiple linear regression analysis showed that baseline DAS28 and changes in DAS28 were easier to predict using CRP and S100A8/9 than baseline SJC and changes in SJC (Tables 2 and 3). The levels of CRP were more important predictors of DAS28 at baseline than the levels of S100A8/9, which were only marginally acceptable as predictors of baseline DAS28 (*P *= 0.052) (Table 2). The levels of both CRP and S100A8/9 at baseline were only weak predictors of the baseline SJC (*P *= 0.045 and *P *= 0.051, respectively). Interestingly, the change in S100A8/9 levels was significantly associated with the change in SJC over time (*P *= 0.001) (Table 3). It can thus be suggested that decreases in serum S100A8/9 over time predict improvements in the number of affected joints.

**Table 2:** Multiple linear regression models for initial DAS28 and changes in DAS28**^a^**

Initial DAS28 (*n *= 43; *r*^2 ^= 0.422; *F *= 16.355; *P *< 0.000)^b^					ΔDAS28 (*n *= 41; *r*^2 ^= 0.349, *F *= 11.727; *P *< 0.000)^c^				
Variables	Parameter estimate	SEM	*t*-value	*P *value	Variable	Parameter estimate	SEM	*t*-value	*P value*
Intercept	4.351	0.246	17.662	0.000	Intercept	-2.117	0.277	-7.649	0.000
Initial CRP	0.037	0.010	3.619	0.001	Initial CRP	0.080	0.038	2.124	0.040
Initial S100A8/9	0.000	0.000	2.007	0.052	ΔCRP	0.136	0.042	3.248	0.002

^a^DAS28, Disease Activity Score for 28 joints; SEM, standard error of the mean; CRP, C-reactive protein. *r*^2 ^values are adjusted. ^b^Excluded predictors are age and initial S100A12. ^c^Excluded predictors are age, initial S100A12, initial S100A8/9, ΔS100A12 and ΔS100A8/9.

**Table 3:** Multiple linear regression models for initial total SJCs and change in SJCs^a^

Initial SJCs (*n *= 43, *r*^2 ^= 0.260, *F *= 8.360; *P *= 0.001)^b^					ΔSJCs (*n *= 41, *r*^2 ^= 0.245, *F *= 13.974; *P *= 0.001)^c^				
Variables	Parameter estimate	SEM	*t*-value	*P *value	Variable	Parameter estimate	SEM	*t*-value	*P *value
Intercept	5.093	1.712	2.975	0.005	Intercept	-5.192	1.148	-3.500	0.001
Initial CRP	0.147	0.071	2.067	0.045	ΔS100A8/9	0.001	0.000	3.738	0.001
Initial S100A8/9	0.001	0.000	2.010	0.051	-	-	-	-	-

^a^SJCs, swollen joint counts; SEM, standard error of the mean; CRP, C-reactive protein. *r*^2 ^values are adjusted. ^b^Excluded predictors are age and initial S100A12. ^c^Excluded predictors are age, initial S100A12, initial S100A8/9, initial CRP, ΔS100A12 and ΔCRP.

## Discussion

To the best of our knowledge, this study is the first to show elevated serum levels of S100 proteins in patients with recent-onset DMARD/GC-naïve RA, the association between S100 proteins and disease activity and normalisation of S100A8/9 in patients who achieve remission after conventional treatment. Furthermore, we have demonstrated that decreases in S100A8/9 levels are associated with clinical improvement in the number of affected joints.

S100 proteins were previously found to be upregulated in the inflamed synovial tissue, synovial fluid and blood of patients with established RA [10-13]. However, in this study, we have shown for the first time significantly increased serum levels of both S100A8/9 and S100A12 proteins in patients with recent-onset RA who had not yet been exposed to conventional treatment. This is consistent with previous reports showing elevated circulating S100 proteins in patients with previously established disease [10-13]. However, compared to previous studies [19,25], the levels of S100A8/9 measured in our study were higher, which can be explained by the use of various antibodies to detect different epitopes in different ELISAs. In our commercial assay, an antibody detecting an epitope which is dependent on S100A8/S100A9 heterocomplex formation and calcium binding was used. Nevertheless, this did not affect the comparative study in general. It has previously been demonstrated that the S100A8/9 and S100A12 proteins show decreased levels in the peripheral circulation in response to different anti-inflammatory therapies [16,17]. Consistent with such findings, we have shown in the present study that the conventional treatment of patients with DMARD/GC-naïve early-stage RA reduces serum levels of S100 proteins to the normal levels found in healthy individuals. As expected, patients with very active disease at baseline had higher levels of S100A8/9 and S100A12 proteins that decreased more significantly as the disease improved than did patients with moderate disease activity. Furthermore, an association between both serum S100 proteins in patients with recent-onset RA is in agreement with previous reports on established RA [26,27], which have indicated that S100A8/9 and S100A12 proteins may be coregulated early in the disease process.

In agreement with previous reports on established RA [10-13,15,20,21], we found an association of both S100A8/9 and S100A12 proteins with laboratory and clinical markers of disease activity in patients with recent-onset RA. Interestingly, this association was more pronounced for S100A8/9 than for S100A12. Higher baseline levels of S100A8/9 predicted decreased CRP levels and improvements in the total number of swollen joints over time. Moreover, decreases in S100A8/9 levels were directly related to clinical and laboratory improvements over time. Multivariate regression analysis revealed that baseline S100A8/9 values were not more predictive of disease activity than traditional biomarkers such as CRP and that they were only marginally acceptable as predictors of baseline disease activity. However, both CRP and S100A8/9 levels were found to be weak predictors for the baseline number of swollen joints. Interestingly, however, changes in the levels of S100A8/9, but not CRP, were associated with changes in the total number of swollen joints over time. It is evident that S100 proteins are extensively produced by activated immune cells of the synovial membrane and synovial fluid in affected joints and pass into the blood circulation [12,13]. Our data support the hypothesis that S100A8/9 protein represents a suitable marker that provides important information about the extent of local inflammation in affected joints, as shown by the strong associations between the decrease in S100A8/9 levels and improvements in swollen joint counts. Although researchers in some studies have found an association between S100 proteins and autoantibodies in patients with RA [15,20,25], we have not confirmed these data, which may be explained by a stronger association with disease activity than with the autoimmune response in the early phase of recent-onset RA. S100A8/9 protein has recently been demonstrated to predict 10-year radiographic progression in patients with established RA [21]. Although our study was not sufficiently long to assess the effect of changes in S100A8/9 levels on the radiographic progression of early RA, it can be suggested that a decrease in serum levels of S100A8/9 over time, which is associated with improvement in the number of affected joints, might be associated with inhibition of further structural joint damage. This remains to be determined in future studies.

## Conclusions

In summary, our data show elevated serum levels of S100 proteins at the onset of RA and a normalisation of S100A8/9 levels in patients who achieved remission shortly after the initiation of conventional treatment. Furthermore, decreases in S100A8/9 rather than CRP levels were associated with improvements in the total number of swollen joints over time. Further studies are needed to determine whether S100A8/9 levels may have a predictive value for further structural damage in patients with recent-onset RA.

## Abbreviations

Anti-CCP: anticyclic citrullinated peptide antibody; CRP: C-reactive protein; DAS28: Disease Activity Score for 28 joints; DMARDs: disease-modifying antirheumatic drugs; ELISA: enzyme-linked immunosorbent assay; ESR: erythrocyte sedimentation rate; EULAR: European League Against Rheumatism; F: female; GC: glucocorticoid; IgM-RF: IgM rheumatoid factor; M: male; MTX: methotrexate; RA: rheumatoid arthritis; SEM: standard error of the mean; SD: standard deviation; SJC: swollen joints count for 66 joints; TNF: tumour necrosis factor; VAS: visual analogue scale.

## Competing interests

The authors declare that they have no competing interests.

## Authors' contributions

LŠ and HM were responsible for the study concept and design. LAC and LP carried out the ELISAs and analysed the data. OP carried out the statistical analysis. LAC, LŠ and OP were responsible for data interpretation and manuscript preparation. JV, LŠ, HM and KP were involved in revising the manuscript and gave their final approval of the version to be published. All authors read and approved the final manuscript.
